# Accommodation for Paying Patients

**Published:** 1919-03-29

**Authors:** 


### HOSPITAL CONSTRUCTION: THE NEW ERA.

### V.—Accommodation for Paying Patients.

FORTY years ago Sir Henry Burdett was engaged in putting through the press his small but well-filled book on the Pay Hospitals of the World. It was an attempt to lay before the English public a summary of the achievements of other countries in the direction of the system advocated and a plea for the removal of what was even in those days a conspicuous abuse of national charity. In both respects the attempt was a success. The summary of Continental and American procedure is exhaustive, and the plea to England, though falling on ears inclined to British deafness, has in the long run gradually come home. Little by little hospitals in all parts of the country have initiated—and with success—the system of receiving in-patients who pay the whole or a part of their cost of maintenance and cure.

To-day owing to various recent causes, directly or indirectly connected with the war, we are faced as a nation with the prospect of a large increase of the paying contingent in our hospitals. These causes are chiefly three. One is the noticeable improvement of the financial prosperity of the wage-earning classes which both enables them to pay and disqualifies them from treatment as paupers; and the other two relate respectively to the man and the woman of the class who in earlier years would always have looked upon a hospital as a place which no "gentleman" or "lady" would willingly enter as a patient. War has, however, changed the hospital from a thing unknown into a thing familiar, and has made it at least seem possible to our upper classes as a blessing open to and desirable for themselves no less than for those who are less fortunate in wealth and position. This development can have but one consequence. The reasonableness of transferring disease and its treatment from the home, where it is a difficulty and danger, to the hospital where it is in its proper place must appeal to all classes.

The paying patient therefore must be expected in greater numbers and must be duly catered for. At the present time in many hospitals there are two classes of paying patients. Those who can afford to pay a substantial fee and are accommodated in private rooms, and those who paying only a small weekly acknowledgment are accorded rather more privileges than the free patients, though they are often mingled with them in wards common to both classes.

The hospital architect, in providing house-room for these two kinds of contributing patients, has a distinct opportunity of improving present arrangements and of avoiding certain defects which are prone to mar the efficiency of his building. We will deal with each class separately, beginning with those who are small payers and are accommodated in wards—that is to say, in rooms containing several beds. It has been the practice to mingle these with other patients in such a way that a ward of, say, twenty beds might contain a dozen free patients, for example, and eight paying patients. This arrangement proves in practice to be very unsatisfactory, not merely because the two classes of patients may each of them slightly resent the presence of the other class on grounds which, though quite illogical, have to be reckoned with, but rather because certain inconveniences arise from thus associating the payers with the non-payers. The former are very naturally granted certain privileges as to visiting hours, etc., which cannot be accorded to the free patients, and the differential treatment in such matters leads to a little ill-feeling. Ill-feeling, however unreasonable, is better avoided in a hospital. It is far more satisfactory, if the number of paying patients can at all be gauged, to provide two or more wards entirely and exclusively for them. Even in a small hospital this can often be done. In a large hospital there need be no difficulty about it whatever.

The single room patient gives a much more awkward problem to the architect. One of the reasons that make it better for a sick man to be treated in a hospital rather than in his home is that in the former he will secure a much better ventilated sick chamber than can ever be provided in a private house. If, therefore, we take him to a hospital only to shut him up in a room which has none of the advantages of cross ventilation and plentiful air space we are rather going back in our progress. In fact the mere provision of a row of rooms measuring ten feet by ten feet, with a window on one side and a door on the other is not good enough. The present writer once designed some private wards in groups of three, so arranged that each room had windows on opposite sides, and so secured cross-current of air. This method of planning though effective is rather extravagant in space. Probably, if the rooms must be along one side (or both sides) of a corridor, the best system for ventilation purposes is not to carry the walls of the room quite up to the ceiling. Assuming the ceiling to be 12 feet 6 inches from the floor, the tops of the walls between one room and the next, and between the rooms and the corridor would be, let us say, 10 feet high. Such an arrangement does not, of course, give sound-proof privacy, but it does give seclusion without producing stuffiness.

A very pleasant arrangement where the conditions of plan admit is so to place the private rooms as to open not into a corridor but into a sunny and airy sitting-room common to the use of such of the occupants of the rooms as are well enough to want the use of a day room. In this case, also, the partitions should be kept sufficiently low to allow the bedrooms to share the benefit of the air and sun space in the sitting-room.

☛ The previous articles appear d on January 25, February 8, March 1 and 15, p. 527.

**The New Era**—(*continued*).

Of course it may be said that the patient who wants privacy does not care for the common sitting-room, and that we want to attract to hospitals persons of the class to whom a boarding-house parlour would be a worse terror than disease. The answer is that, with the growth of the paying patient movement there is no reason why hospitals of the future should not provide all that any patient wants and can pay for. The only limit is placed by the laws of hygiene. Provided that the demands of the richer private patient are merely for such architectural luxuries as will contribute to his health of body and mind, there can surely be no reason why he should not pay for and get them as well in a hospital as in a home or a hotel. We may thus arrive at the construction of private suites, each with its sitting-room, bathroom, and sanitary needs connected. The arrangement prevails in America, but there appears to be less care taken in the States to isolate the sanitary group than would be acceptable to English notions of ventilation.

